# Interaction of scales for a singularly perturbed degenerating nonlinear Robin problem

**DOI:** 10.1098/rsta.2022.0159

**Published:** 2022-11-14

**Authors:** Paolo Musolino, Gennady Mishuris

**Affiliations:** ^1^ Dipartimento di Scienze Molecolari e Nanosistemi, Università Ca’ Foscari Venezia, via Torino 155, 30172 Venezia Mestre, Italy; ^2^ Department of Mathematics, Aberystwyth University, Ceredigion, Aberystwyth SY23 3BZ, UK

**Keywords:** singularly perturbed boundary value problem, Laplace equation, nonlinear Robin condition, perforated domain, integral equations

## Abstract

We study the asymptotic behaviour of solutions of a boundary value problem for the Laplace equation in a perforated domain in $R^{n}$, n≥3, with a (nonlinear) Robin boundary condition on the boundary of the small hole. The problem we wish to consider degenerates in three respects: in the limit case, the Robin boundary condition may degenerate into a Neumann boundary condition, the Robin datum may tend to infinity, and the size ϵ of the small hole where we consider the Robin condition collapses to 0. We study how these three singularities interact and affect the asymptotic behaviour as ϵ tends to 0, and we represent the solution and its energy integral in terms of real analytic maps and known functions of the singular perturbation parameters.

This article is part of the theme issue ‘Non-smooth variational problems and applications’.

## 1. Introduction

This article is devoted to the study of the asymptotic behaviour of solutions of a boundary value problem for the Laplace equation in a perforated domain in $R^{n}$, n≥3, with a (nonlinear) Robin boundary condition which degenerates into a Neumann condition on the boundary of the small hole. The problem we wish to consider degenerates in three respects. First, in the limit case, the Robin boundary condition may degenerate into a Neumann boundary condition (i.e. the coefficient of the trace of the solution in the boundary condition may vanish). Second, the Robin datum may tend to infinity. Finally, the size ϵ of the small hole where we consider the Robin condition approaches the degenerate value ϵ=0.

The behaviour of solutions to boundary value problems with degenerating or perturbed boundary conditions has been studied by many authors. A family of Poincaré problems approximating a mixed boundary value problem for the Laplace equation in the plane has been studied by Wendland *et al.* [1]. A study of the convergence of the solution of the Helmholtz equation with boundary condition of the type −ϵ(∂u/∂ν)+u=g to the solution with Dirichlet condition u=g as ϵ→0 can be found in the work of Kirsch [2]. Costabel and Dauge [3] studied a mixed Neumann–Robin problem for the Laplace operator, where the Robin condition tends to a Dirichlet condition as the perturbation parameter tends to 0. Boundary value problems for Maxwell equations with singularly perturbed boundary conditions have been analysed, for instance, by Ammari and Nédélec [4]. Also, singularly perturbed transmission problems have been investigated by Schmidt and Hiptmair [5] by means of integral equation methods. Dalla Riva and Mishuris [6] have investigated the solvability of a small nonlinear perturbation of a homogeneous linear transmission problem by using potential-theoretical techniques. The present article represents a continuation of the analysis done in [7], where the authors considered the behaviour as δ→0 of the solutions to the boundary value problem

1.1 $$\left\{ \begin{array}{ll} \Delta u(x)=0 & \forall \;x\in \Omega^{o}\setminus \bar{\Omega^{i}},\\ \frac{\partial }{\partial \nu_{\Omega^{o}}}u(x)=g^{o}(x) & \forall \;x\in \partial \Omega^{o},\\ \frac{\partial }{\partial \nu_{\Omega^{i}}}u(x)=\delta F_{\delta }(u(x))+g^{i}(x) & \forall \;x\in \partial \Omega^{i}, \end{array}\right.$$

where $\Omega^{o}$ and $\Omega^{i}$ are sufficiently regular bounded open sets such that $\bar{\Omega^{i}}\subseteq \Omega^{o}$. In this equation, the superscript ‘o’ stands for ‘outer domain’ and the superscript ‘i’ stands for ‘inner domain.’ The problem generalizes a linear problem that, under suitable assumptions, admits a unique solution $u_{\delta }$ for each δ>0. When δ=0, the problem degenerates into the Neumann problem

1.2 $$\left\{ \begin{array}{ll} \Delta u(x)=0 & \forall \;x\in \Omega^{o}\setminus \bar{\Omega^{i}},\\ \frac{\partial }{\partial \nu_{\Omega^{o}}}u(x)=g^{o}(x) & \forall \;x\in \partial \Omega^{o},\\ \frac{\partial }{\partial \nu_{\Omega^{i}}}u(x)=g^{i}(x) & \forall \;x\in \partial \Omega^{i}. \end{array}\right.$$

As is well known, this Neumann problem may have infinite solutions or no solutions, depending on compatibility conditions on the Neumann datum. In [7] we proved that, under suitable assumptions, solutions to (1.1) exist and that they diverge if the compatibility condition on the Neumann datum for the existence of solutions to (1.2) does not hold. In [7], we considered a Robin problem as a simplified model for the transmission problem for a composite domain with imperfect (non-natural) conditions along the joint boundary. Such nonlinear transmission conditions frequently appear in practical applications for various nonlinear multiphysics problems (e.g. [8–15]). All such transmission conditions have been derived using formal variational or asymptotic techniques (see e.g. [16–18]). However, accurate analysis of their solvability and solution regularity has not been performed. One of the aims of the present paper is to address this need. On the other hand, the problem in question, though simpler than most of those arising in applications, is rich enough as it contains some features influencing the final result. This refers not only to the condition itself but also the surface on which they hold.

In [7], we considered the case where the surface on which we consider the Robin condition is the boundary of a fixed hole $\Omega^{i}$. Here, we wish to study the case where the hole becomes small and degenerates into a point. Then a natural question arises: if we replace the set $\Omega^{i}$ by a small set $\epsilon \omega^{i}$ (with ϵ close to 0) and the parameter δ by a function δ(ϵ) possibly tending to zero as ϵ→0, what happens? How does the geometric degeneracy (the set $\epsilon \omega^{i}$ collapsing to the origin when ϵ=0) interact with the possible degeneracy of the boundary condition if δ(ϵ)→0 as ϵ approaches 0? We also observe that even though several techniques are available for the analysis of linear problems, the presence of a nonlinear boundary condition requires a specific type of analysis since, for example, existence and uniqueness of solutions is not immediately ensured.

The purpose of the present article is to give answers to these questions. Here we consider only the case of dimension n≥3. Indeed, our technique is based on potential theory, and the two-dimensional case requires a specific analysis due to different aspects of the fundamental solution of the Laplacian. In particular, if n=2 the fundamental solution $S_{n}$ of the Laplacian equals (log⁡|x|)/(2π), whereas if n≥3 the fundamental solution $S_{n}$ is a multiple of $1/|x{|}^{n-2}$. This leads to different rescaling behaviour of $S_{n}(\epsilon x)$ and to different behaviour at infinity of single-layer potentials, which are among our main tools in the analysis. We note that the set $\epsilon \omega^{i}$ when ϵ is close to zero can be seen as a small hole in the set $\Omega^{o}$. The behaviour of the solutions to boundary value problems in domains with small holes has long been investigated by the expansion methods of asymptotic analysis. Such methods are mainly based on elliptic theory and allow the treatment of a large variety of linear problems. As examples, we mention the method of matching outer and inner asymptotic expansions of Il’in [19] and the compound asymptotic expansion method of Maz’ya *et al.* [20,21], which allows the treatment of general Douglis–Nirenberg elliptic boundary value problems in domains with perforations and corners. More recently, Maz’ya *et al.* [22] provided asymptotic analysis of Green’s kernels in domains with small cavities by applying the method of mesoscale asymptotic approximations (see also the papers [23–27]). Moreover, we refer to Ammari and Kang [28] for several applications to inverse problems and Novotny and Sokołowski [29] for applications to topological optimization.

Instead of the methods of asymptotic analysis, here we exploit the so-called functional-analytic approach proposed by Lanza de Cristoforis in [30]. The goal of this approach is to represent solutions to problems in perturbed domains in terms of real analytic maps and known functions of the perturbation parameter. For a detailed presentation of the functional-analytic approach, we refer to Dalla Riva *et al.* [31]. Here, we mention that the functional-analytic approach has been used to analyse a nonlinear Robin problem for the Laplace equation by Lanza de Cristoforis [32] and Lanza de Cristoforis and Musolino [33] and to analyse nonlinear traction problems for Lamé equations by, for example, Dalla Riva and Lanza de Cristoforis [34–36] and Falconi *et al.* [37].

As a first step, we introduce the geometric setting in which we are going to consider our boundary value problem. As the dimensional parameter, we take a natural number

n∈N∖{0,1,2}.

Then, to define the perforated domain, we consider a regularity parameter α∈(0,1) and two subsets $\omega^{i}$ and $\Omega^{o}$ of $R^{n}$ satisfying the following condition:

$$\begin{array}{rl} & \omega^{i}\;\text{and }\Omega^{o}\;\text{are bounded open connected subsets of }R^{n}\;\text{of class }C^{1,\alpha }\\ & \text{such that }0\in \Omega^{o}\cap \omega^{i}\;\text{and both }R^{n}\setminus \bar{\omega^{i}}\;\text{and }R^{n}\setminus \bar{\Omega^{o}}\;\text{are connected}. \end{array}$$

The set $\Omega^{o}$ plays the role of the unperturbed domain, whereas the set $\omega^{i}$ represents the shape of the perforation. We refer, for instance, to Gilbarg and Trudinger [38] for the definition of sets and functions of the Schauder class $C^{k,\alpha }$ (k∈N). We fix

$$\epsilon_{0}\equiv \text{sup}\{\theta \in (0,+\infty ):\epsilon \bar{\omega^{i}}\subseteq \Omega^{o}\;\forall \epsilon \in (-\theta ,\theta )\}.$$

We note that if $\epsilon \in (0,\epsilon_{0})$, the set $\epsilon \bar{\omega^{i}}$ (which we think as a hole) is contained in $\Omega^{o}$ and therefore we can remove it from the unperturbed domain. We define the perforated domain Ω(ϵ) by setting

$$\Omega (\epsilon )\equiv \Omega^{o}\setminus \epsilon \bar{\omega^{i}}$$

for all $\epsilon \in (0,\epsilon_{0})$. When ϵ approaches zero, the set Ω(ϵ) degenerates to the punctured domain $\Omega^{o}\setminus \{0\}$. Clearly, the boundary ∂Ω(ϵ) of Ω(ϵ) consists of the two connected components $\partial \Omega^{o}$ and $\partial (\epsilon \omega^{i})=\epsilon \partial \omega^{i}$. Therefore we can identify, for example, $C^{0,\alpha }(\partial \Omega (\epsilon ))$ with the product $C^{0,\alpha }(\partial \Omega^{o})\times C^{0,\alpha }(\epsilon \partial \omega^{i})$. Moreover, after a suitable rescaling, we can identify functions in $C^{0,\alpha }(\epsilon \partial \omega^{i})$ with functions in $C^{0,\alpha }(\partial \omega^{i})$. Having introduced the geometric aspects of our problem, we need to define the boundary data. To do this, we fix two functions

$$g^{o}\in C^{0,\alpha }(\partial \Omega^{o})\;\mathrm{and}\;g^{i}\in C^{0,\alpha }(\partial \omega^{i}).$$

Then we take a family ${\left\{F_{\epsilon }\right\}}_{\epsilon \in ]0,\epsilon_{0}[}$ of functions from R to R and two functions δ(⋅) and ρ(⋅) from $\left(0,\epsilon_{0}\right)$ to (0,+∞). As we shall see, the function $g^{o}$ represents the Neumann datum on the exterior boundary $\partial \Omega^{o}$. The family of functions ${\left\{F_{\epsilon }\right\}}_{\epsilon \in ]0,\epsilon_{0}[}$ will allow us to define the nonlinear Robin condition on $\epsilon \partial \omega^{i}$, and δ(ϵ) will be the coefficient of a function of the Dirichlet trace in the Robin condition. We will consider a non-homogeneous Robin condition, and thus the corresponding datum will be $g^{i}(\cdot /\epsilon )/\rho (\epsilon )$. Next, for each $\epsilon \in (0,\epsilon_{0})$, we want to consider a nonlinear boundary value problem for the Laplace operator. Namely, we consider a Neumann condition on $\partial \Omega^{o}$ and a nonlinear Robin condition on $\epsilon \partial \omega^{i}$. Thus, for each $\epsilon \in (0,\epsilon_{0})$ we consider the following problem:

1.3 $$\left\{ \begin{array}{ll} \Delta u(x)=0 & \forall \;x\in \Omega (\epsilon ),\\ \frac{\partial }{\partial \nu_{\Omega^{o}}}u(x)=g^{o}(x) & \forall \;x\in \partial \Omega^{o},\\ \frac{\partial }{\partial \nu_{\epsilon \omega^{i}}}u(x)=\delta (\epsilon )F_{\epsilon }(u(x))+\frac{g^{i}(x/\epsilon )}{\rho (\epsilon )} & \forall \;x\in \epsilon \partial \omega^{i}, \end{array}\right.$$

where $\nu_{\Omega^{o}}$ and $\nu_{\epsilon \omega^{i}}$ denote the outward unit normals to $\partial \Omega^{o}$ and to $\partial (\epsilon \omega^{i})$, respectively. Our aim is to analyse the behaviour of the solutions to problem (1.3) as ϵ→0. As we have already mentioned, when ϵ tends to 0 the hole $\epsilon \omega^{i}$ degenerates into the origin 0. Moreover, if δ(ϵ) tends to 0 as ϵ→0, the Robin condition may degenerate into a Neumann condition. Furthermore, we will also allow the term ρ(ϵ) to tend to 0, which may generate a further singularity. An aspect we wish to highlight in the present article is how all these singularities interact together. Our main results are represented by theorems 4.4 and 4.6, which describe in detail the asymptotic behaviour of the solutions as ϵ→0, and theorem 4.7, which concerns the behaviour of the energy integrals of the solutions. These results highlight the interactions of different scales. Moreover, as we will see, it will be crucial to assume that the quantities ϵδ(ϵ) and $\epsilon^{n-1}/\rho (\epsilon )$ have limits as ϵ→0. Incidentally, we observe that interactions of scales are well known to possibly cause strange phenomena in the limiting behaviour of solutions. As an example, we mention the celebrated works of Cioranescu and Murat [39,40] and of Marčenko and Khruslov [41], as well as the more recent articles of Arrieta and Lamberti [42], Arrieta *et al.* [43] and Ferraresso and Lamberti [44]. We also mention the work of Bonnetier *et al.* [45] concerning small perturbations in the type of boundary conditions and of Felli *et al.* [46,47] on disappearing Neumann or Dirichlet regions in mixed eigenvalue problems.

We observe that in the present article, the boundary of the hole depends on ϵ simply through a dilation. However, in the literature one can find examples where the geometry changes in a more drastic way, as in the case of oscillating boundaries (see e.g. [42–44]). On the other hand, one may also consider the case where the geometry is fixed and the boundary condition is changing, as in [7].

This article is organized as follows. In §2, we analyse a toy problem in an annular domain. In §3, we transform problem (1.3) into an equivalent system of integral equations. In §4, we analyse this system and prove our main results on the asymptotic behaviour of a family of solutions and the corresponding energy integrals.

## 2. A toy problem

As we have done in [7], we consider problem (1.3) in the annular domain

$$\Omega (\epsilon )\equiv B_{n}(0,1)\setminus \bar{B_{n}(0,\epsilon )}=B_{n}(0,1)\setminus \epsilon \bar{B_{n}(0,1)},$$

i.e. we take $\Omega^{o}\equiv B_{n}(0,1)$ and $\omega^{i}\equiv B_{n}(0,1)$, where, for r>0, the symbol $B_{n}(0,r)$ denotes the open ball in $R^{n}$ of centre 0 and radius r. We will then set $\epsilon_{0}=1$, $F_{\epsilon }(\tau )=\tau$ for all τ∈R and all $\epsilon \in (0,\epsilon_{0})$, $g^{o}=a$ and $g^{i}=b$, where a,b∈R. Moreover, we consider two functions δ,ρ:(0,1)→(0,+∞). Then for each ϵ∈(0,1) we consider the problem

2.1 $$\left\{ \begin{array}{ll} \Delta u(x)=0 & \forall \;x\in B_{n}\;(0,1)\setminus \bar{B_{n}(0,\epsilon )},\\ \frac{\partial }{\partial \nu_{B_{n}(0,1)}}u(x)=a & \forall \;x\in \partial B_{n}\;(0,1),\\ \frac{\partial }{\partial \nu_{B_{n}(0,\epsilon )}}u(x)=\delta (\epsilon )u(x)+\frac{b}{\rho (\epsilon )} & \forall \;x\in \partial B_{n}\;(0,\epsilon ). \end{array}\right.$$

As is well known, for each ϵ∈(0,1) a solution $u_{\epsilon }\in C^{1,\alpha }(\bar{\Omega (\epsilon )})$ to problem (2.1) exists and is unique (see [31], theorem 6.56). On the other hand, if instead we put ϵ=0 in (2.1), the hole disappears and we are led to consider the Neumann problem

2.2 $$\left\{ \begin{array}{ll} \Delta u(x)=0 & \forall \;x\in B_{n}(0,1),\\ \frac{\partial }{\partial \nu_{B_{n}(0,1)}}u(x)=a & \forall \;x\in \partial B_{n}(0,1). \end{array}\right.$$

As with any Neumann problem, the solvability of (2.2) is subject to compatibility conditions on the Neumann datum on $\partial B_{n}(0,1)$. In this specific case of constant Neumann datum, problem (2.2) has a solution if and only if a=0. Obviously, if a=0, then the Neumann problem (2.2) has a one-dimensional space of solutions, which consists of the space of constant functions in $\bar{B_{n}(0,1)}$; if instead a≠0, problem (2.2) does not have any solution. On the other hand, if one considers the behaviour of the unique solution $u_{\epsilon }$ of problem (2.1), the earlier remark clearly implies that in general $u_{\epsilon }$ cannot converge to a solution of (2.2) as ϵ→0 if the compatibility condition a=0 does not hold. Also, even if a=0, we shall see that the solutions may diverge as ϵ→0, depending on the behaviour of the functions δ(ϵ) and ρ(ϵ) for ϵ close to 0. Moreover, one would like to understand how the behaviour of δ(ϵ) and ρ(ϵ) affects the asymptotic behaviour of $u_{\epsilon }$ and whether there is a ‘memory’ of the Robin condition. In the specific case of our annular domain and constant data, we can construct explicitly the solution $u_{\epsilon }$. Then we try to understand the behaviour of $u_{\epsilon }$ as ϵ→0. To construct explicitly $u_{\epsilon }$, we search for a solution of (2.1) in the form

$$u_{\epsilon }(x)\equiv A_{\epsilon }\frac{1}{(2-n)|x{|}^{n-2}}+B_{\epsilon }\;\forall \;x\in \bar{\Omega (\epsilon )},$$

with $A_{\epsilon }$ and $B_{\epsilon }$ chosen so that the boundary conditions of problem (2.1) are satisfied. By a straightforward computation, we must have

2.3 $$u_{\epsilon }(x)\equiv a\frac{1}{(2-n)|x{|}^{n-2}}+\frac{1}{\delta (\epsilon )}(\frac{a}{\epsilon^{n-1}}-\frac{b}{\rho (\epsilon )})+\frac{a}{(n-2)\epsilon^{n-2}}\;\forall \;x\in \bar{\Omega (\epsilon )}.$$

We now note that we can rewrite equation (2.3) as

2.4 $$u_{\epsilon }(x)\equiv a\frac{1}{(2-n)|x{|}^{n-2}}+\frac{1}{\delta (\epsilon )\epsilon^{n-1}}(a-b\;\frac{\epsilon^{n-1}}{\rho (\epsilon )}+\frac{a}{\left(n-2\right)}\;\delta (\epsilon )\epsilon )\;\forall \;x\in \bar{\Omega (\epsilon )}.$$

In particular, if $d_{0}\equiv \lim_{\epsilon \to 0}\epsilon \delta (\epsilon )\in R$, $r_{0}\equiv \lim_{\epsilon \to 0}\epsilon^{n-1}/\rho (\epsilon )\in R$ and $a-br_{0}+a\;d_{0}/(n-2)\neq 0$, then $u_{\epsilon }(x)$ is asymptotic to $\left(a-br_{0}+ad_{0}/(n-2))/(\epsilon^{n-1}\delta (\epsilon )\right)$ as ϵ tends to 0, when x is fixed in $\bar{B_{n}(0,1)}\setminus \{0\}$. In conclusion, under suitable assumptions on the behaviour of δ(ϵ) and ρ(ϵ) as ϵ→0, we see that the value of the solution $u_{\epsilon }$ at a fixed point $x\in \bar{B_{n}(0,1)}\setminus \{0\}$ behaves like $1/(\epsilon^{n-1}\delta (\epsilon ))$ and there is some sort of interaction of scales influencing the limiting behaviour of the solution. Similarly, if one considers the energy integral of $u_{\epsilon }$, a direct computation shows that

2.5 $$\begin{array}{rl} \int_{\Omega (\epsilon )}|\nabla u_{\epsilon }(x){|}^{2}\;dx & \;=\int_{\Omega (\epsilon )}{\left|\nabla \left(a\frac{1}{(2-n)|x{|}^{n-2}}\right)\right|}^{2}\;dx=\int_{\Omega (\epsilon )}a^{2}\frac{1}{|x{|}^{2n-2}}\;dx\\ & \;=a^{2}s_{n}\int_{\epsilon }^{1}\frac{1}{r^{n-1}}\;dr=a^{2}\frac{s_{n}}{\left(n-2\right)}\frac{1}{\epsilon^{n-2}}(1-\epsilon^{n-2}), \end{array}$$

where the symbol $s_{n}$ denotes the (n−1)-dimensional measure of $\partial B_{n}(0,1)$. In particular, if a≠0, the energy integral of the solution $\int_{\Omega (\epsilon )}|\nabla u_{\epsilon }(x){|}^{2}\;dx$ behaves like $1/\epsilon^{n-2}$.

Our aim is to recover and understand such behaviour of the solution and of its energy integral in a more general situation, for both the geometry and the boundary conditions. Indeed, we will show that the main features discussed above can be identified in the general solution (compare (2.4) with (4.14) and (2.5) with (4.17)). We emphasize that one can derive a uniform asymptotic solution by the methods of [20,21,26]. In particular, one can identify the uniform limit far from the hole as a first approximation. Then one can correct such a limit in order to improve the approximation on rescaled sets and repeat the procedure in order to reduce the error. We will show in remarks 4.2 and 4.5 how one can deduce the above considerations from the results of §4 (which can thus be seen as analogous formulas in more general settings).

## 3. An integral equation formulation of the boundary value problem

As in [7], to analyse problem (1.3) for ϵ close to 0, we exploit the so-called functional-analytic approach (see [31]). This method is based on classical potential theory, which allows one to obtain an integral equation formulation of (1.3). First we need to introduce some notation. We denote by $S_{n}$ the function from $R^{n}\setminus \{0\}$ to R defined by

$$S_{n}(x)\equiv \frac{1}{(2-n)s_{n}|x{|}^{n-2}}\;\forall \;x\in R^{n}\setminus \{0\}.$$

Since n≥3, as is well known, $S_{n}$ is a fundamental solution of the Laplace operator. By means of the fundamental solution $S_{n}$, we construct some integral operators (namely, single-layer potentials) that we use to represent harmonic functions (and thus, in particular, the solutions of problem (1.3)). So let Ω be a bounded open connected subset of $R^{n}$ of class $C^{1,\alpha }$. If $\mu \in C^{0}(\partial \Omega )$, we introduce the single-layer potential by setting

$$v[\partial \Omega ,\mu ](x)\equiv \int_{\partial \Omega }S_{n}(x-y)\mu (y)\;d\sigma_{y}\;\forall \;x\in R^{n},$$

where dσ denotes the area element of a manifold imbedded in $R^{n}$. It is well known that if $\mu \in C^{0}(\partial \Omega )$, then v[∂Ω,μ] is continuous in $R^{n}$. Moreover, if $\mu \in C^{0,\alpha }(\partial \Omega )$, then the function $v^{+}[\partial \Omega ,\mu ]\equiv v[\partial \Omega ,\mu ]{|}_{\bar{\Omega }}$ belongs to $C^{1,\alpha }(\bar{\Omega })$ and the function $v^{-}[\partial \Omega ,\mu ]\equiv v[\partial \Omega ,\mu ]{|}_{R^{n}\setminus \Omega }$ belongs to $C_{\mathrm{loc}}^{1,\alpha }(R^{n}\setminus \Omega )$. The normal derivative of the single-layer potential on ∂Ω, on the other hand, exhibits a jump. To describe the jump, we set

$$W^{*}[\partial \Omega ,\mu ](x)\equiv \int_{\partial \Omega }\nu_{\Omega }(x)\cdot \nabla S_{n}(x-y)\mu (y)\;d\sigma_{y}\;\forall \;x\in \partial \Omega ,$$

where $\nu_{\Omega }$ denotes the outward unit normal to ∂Ω. If $\mu \in C^{0,\alpha }(\partial \Omega )$, the function $W^{*}[\partial \Omega ,\mu ]$ belongs to $C^{0,\alpha }(\partial \Omega ),$ and we have

$$\frac{\partial }{\partial \nu_{\Omega }}v^{\pm }[\partial \Omega ,\mu ]=\mp \frac{1}{2}\mu +W^{*}[\partial \Omega ,\mu ]\;\;\text{on }\partial \Omega .$$

As we shall see in lemma 3.1, to represent the functions on $\bar{\Omega (\epsilon )}$ which are harmonic and satisfy the boundary conditions, we will exploit single-layer potentials having densities with zero integral mean on $\partial \Omega^{o}$ plus constants. Therefore, we find it convenient to set

$$C^{0,\alpha }{\left(\partial \Omega^{o}\right)}_{0}\equiv \left\{f\in C^{0,\alpha }(\partial \Omega^{o}):\int_{\partial \Omega^{o}}f\;d\sigma =0\right\}.$$

More precisely, in lemma 3.1 we represent a function $u\in C^{1,\alpha }(\bar{\Omega (\epsilon )})$ such that Δu=0 in Ω(ϵ) as a single-layer potential plus the ϵ-dependent constant $\xi /(\delta (\epsilon )\epsilon^{n-1})$. The reason for the choice of such a constant is that in view of the results of §2, we expect the presence of a constant behaving like $1/(\delta (\epsilon )\epsilon^{n-1})$ as ϵ→0 in the representation formula for the solutions of (1.3). The proof of lemma 3.1 can be deduced from classical potential theory (cf. Folland [48], ch. 3, and Dalla Riva *et al.* [31], proof of proposition 6.49).

**Lemma 3.1.**

*Let* $\epsilon \in (0,\epsilon_{0})$. *Let* $u\in C^{1,\alpha }(\bar{\Omega (\epsilon )})$ *be such that* Δu=0 *in* Ω(ϵ). *Then there exists a unique triple* $(\mu^{o},\mu^{i},\xi )\in C^{0,\alpha }{\left(\partial \Omega^{o}\right)}_{0}\times C^{0,\alpha }(\partial \omega^{i})\times R$ *such that*

$$u(x)=\int_{\partial \Omega^{o}}S_{n}(x-y)\mu^{o}(y)\;d\sigma_{y}+\int_{\partial \omega^{i}}S_{n}(x-\epsilon s)\mu^{i}(s)\;d\sigma_{s}+\frac{\xi }{\delta (\epsilon )\epsilon^{n-1}}\;\forall \;x\in \bar{\Omega (\epsilon )}.$$

By exploiting lemma 3.1, we can establish a correspondence between the solutions of problem (1.3) and those of a (nonlinear) system of integral equations.

**Proposition 3.2.**

*Let* $\epsilon \in (0,\epsilon_{0})$. *Then the map from the set of triples* $(\mu^{o},\mu^{i},\xi )\in C^{0,\alpha }{\left(\partial \Omega^{o}\right)}_{0}\times C^{0,\alpha }(\partial \omega^{i})\times R$ *such that*

3.1 $$\begin{array}{rl} & \;-\frac{1}{2}\mu^{o}(x)+\int_{\partial \Omega^{o}}\nu_{\Omega^{o}}(x)\cdot \nabla S_{n}(x-y)\mu^{o}(y)\;d\sigma_{y}\\ & \;\;+\int_{\partial \omega^{i}}\nu_{\Omega^{o}}(x)\cdot \nabla S_{n}(x-\epsilon s)\mu^{i}(s)\;d\sigma_{s}=g^{o}(x)\;\forall \;x\in \partial \Omega^{o} \end{array}$$

*and*

3.2 $$\begin{array}{rl} & \frac{1}{2}\mu^{i}(t)+\epsilon^{n-1}\int_{\partial \Omega^{o}}\nu_{\omega^{i}}(t)\cdot \nabla S_{n}(\epsilon t-y)\mu^{o}(y)\;d\sigma_{y}+\int_{\partial \omega^{i}}\nu_{\omega^{i}}(t)\cdot \nabla S_{n}(t-s)\mu^{i}(s)\;d\sigma_{s}\\ & \;=\epsilon^{n-1}\delta (\epsilon )F_{\epsilon }(\int_{\partial \Omega^{o}}S_{n}(\epsilon t-y)\mu^{o}(y)\;d\sigma_{y}+\frac{1}{\epsilon^{n-2}}\int_{\partial \omega^{i}}S_{n}(t-s)\mu^{i}(s)\;d\sigma_{s}\\ & \;\;\;\;\;\;\;+\frac{\xi }{\delta (\epsilon )\epsilon^{n-1}})+g^{i}(t)\frac{\epsilon^{n-1}}{\rho (\epsilon )}\;\forall \;t\in \partial \omega^{i} \end{array}$$

*to the set of functions* $u\in C^{1,\alpha }(\bar{\Omega (\epsilon )})$ *that solve problem* (1.3), *which takes a triple* $\left(\mu^{o},\mu^{i},\xi \right)$ *to*

3.3 $$\int_{\partial \Omega^{o}}S_{n}(x-y)\mu^{o}(y)\;d\sigma_{y}+\int_{\partial \omega^{i}}S_{n}(x-\epsilon s)\mu^{i}(s)\;d\sigma_{s}+\frac{\xi }{\delta (\epsilon )\epsilon^{n-1}}\;\forall \;x\in \bar{\Omega (\epsilon )},$$

*is a bijection*.

**Proof.**

If $(\mu^{o},\mu^{i},\xi )\in C^{0,\alpha }{\left(\partial \Omega^{o}\right)}_{0}\times C^{0,\alpha }(\partial \omega^{i})\times R$, then we know that the function in (3.3) belongs to $C^{1,\alpha }(\bar{\Omega (\epsilon )})$ and is harmonic in Ω(ϵ). Moreover, if $\left(\mu^{o},\mu^{i},\xi \right)$ satisfies system (3.1)–(3.2), then the jump formula for the normal derivative of the single-layer potential implies the validity of the boundary condition in problem (1.3). Hence, the function in (3.3) solves problem (1.3).

Conversely, if $u\in C^{1,\alpha }(\bar{\Omega (\epsilon )})$ satisfies (1.3), then lemma 3.1 for harmonic functions ensures that there exists a unique triple $(\mu^{o},\mu^{i},\xi )\in C^{0,\alpha }{\left(\partial \Omega^{o}\right)}_{0}\times C^{0,\alpha }(\partial \omega^{i})\times R$ such that

$$u(x)=\int_{\partial \Omega^{o}}S_{n}(x-y)\mu^{o}(y)\;d\sigma_{y}+\int_{\partial \omega^{i}}S_{n}(x-\epsilon s)\mu^{i}(s)\;d\sigma_{s}+\frac{\xi }{\delta (\epsilon )\epsilon^{n-1}}\;\forall \;x\in \bar{\Omega (\epsilon )}.$$

Then the formula for the normal derivative of a single-layer potential and the boundary conditions in (1.3) imply that (3.1) and (3.2) are satisfied. Hence, the map of the statement is a bijection.

Now that the correspondence between the solutions of boundary value problem (1.3) and those of the system of integral equations (3.1)–(3.2) is established, we wish to study the behaviour of the solutions to system (3.1)–(3.2) as ϵ→0. Note that if $\epsilon \in (0,\epsilon_{0})$, we can write

$$\begin{array}{rl} \epsilon^{n-1}\delta (\epsilon )F_{\epsilon }(\int_{\partial \Omega^{o}}S_{n}(\epsilon t-y) & \mu^{o}(y)\;d\sigma_{y}+\frac{1}{\epsilon^{n-2}}\int_{\partial \omega^{i}}S_{n}(t-s)\mu^{i}(s)\;d\sigma_{s}+\frac{\xi }{\delta (\epsilon )\epsilon^{n-1}})\\ =\epsilon^{n-1}\delta (\epsilon )F_{\epsilon }(\frac{1}{\epsilon^{n-1}\delta (\epsilon )}( & \epsilon^{n-1}\delta (\epsilon )\int_{\partial \Omega^{o}}S_{n}(\epsilon t-y)\mu^{o}(y)\;d\sigma_{y}\\ & \;+\epsilon \delta (\epsilon )\int_{\partial \omega^{i}}S_{n}(t-s)\mu^{i}(s)\;d\sigma_{s}+\xi ))\;\forall \;t\in \partial \omega^{i}. \end{array}$$

We now wish to analyse equation (3.2) for ϵ small. As in [7], we need to make a structural assumption on the nonlinearity, i.e. on the family of functions $R∋\tau \mapsto \epsilon^{n-1}\delta (\epsilon )F_{\epsilon }(\tau /(\epsilon^{n-1}\delta (\epsilon )))$ for ϵ close to 0. So we assume the following:

3.4 $$\begin{array}{rl} & \text{there exist }\epsilon_{1}\in (0,\epsilon_{0}),\;m\in N,\text{ a real analytic function }\tilde{F}:R^{m+1}\to R\\ & \text{and a function }\eta :(0,\epsilon_{1})\to R^{m}\text{ such that }\eta_{0}=\lim_{\epsilon \to 0}\eta (\epsilon )\in R^{m}\text{ and }\\ & \epsilon^{n-1}\delta (\epsilon )F_{\epsilon }\left(\frac{1}{\epsilon^{n-1}\delta (\epsilon )}\tau \right)=\tilde{F}(\tau ,\eta (\epsilon ))\;\text{for all }(\tau ,\epsilon )\in R\times (0,\epsilon_{1}). \end{array}$$

As a simple example, one can take as $F_{\epsilon }$ a small polynomial perturbation of the identity. For example,

$$F_{\epsilon }(z)=z+h(\epsilon )z^{m},$$

where m∈N∖{0,1} and h is a certain function from $\left(0,\epsilon_{1}\right)$ to R. Then we have

$$\epsilon^{n-1}\delta (\epsilon )F_{\epsilon }(\frac{1}{\epsilon^{n-1}\delta (\epsilon )}\tau )=\tau +\frac{h(\epsilon )}{{\left(\epsilon^{n-1}\delta (\epsilon )\right)}^{m-1}}\tau^{m}.$$

If

$$\lim_{\epsilon \to 0}\frac{h(\epsilon )}{{\left(\epsilon^{n-1}\delta (\epsilon )\right)}^{m-1}}\in R,$$

then one has

$$\eta (\epsilon )=\frac{h(\epsilon )}{{\left(\epsilon^{n-1}\delta (\epsilon )\right)}^{m-1}}\;\text{and}\;\tilde{F}(\tau ,\eta (\epsilon ))=\tau +\eta (\epsilon )\tau^{m}=\tau +\frac{h(\epsilon )}{{\left(\epsilon^{n-1}\delta (\epsilon )\right)}^{m-1}}\tau^{m}.$$

On the other hand, one could also construct $F_{\epsilon }$ starting from a given $\tilde{F}$ and η(ϵ). This would allow one to generate more involved nonlinearities (if perhaps less natural).

Here, we observe that different structures of the nonlinearity may be tackled by modifying our approach. Although the type of nonlinearity we consider is quite specific, we emphasize that our techniques are not confined to linear boundary conditions and apply also in some nonlinear cases. At the same time, we are also interested in the linear case, since the degeneracy appears there as well. Therefore, for us, it is enough to include some (nonlinear) perturbations of the linear case.

## 4. Analytic representation formulas for the solution of the boundary value problem

We observe that under the additional assumption (3.4), equations (3.1) and (3.2) take the forms

4.1 $$\begin{array}{rl} & \;-\frac{1}{2}\mu^{o}(x)+\int_{\partial \Omega^{o}}\nu_{\Omega^{o}}(x)\cdot \nabla S_{n}(x-y)\mu^{o}(y)\;d\sigma_{y}\\ & \;\;+\int_{\partial \omega^{i}}\nu_{\Omega^{o}}(x)\cdot \nabla S_{n}(x-\epsilon s)\mu^{i}(s)\;d\sigma_{s}=g^{o}(x)\;\forall \;x\in \partial \Omega^{o} \end{array}$$

and

4.2 $$\begin{array}{rl} & \frac{1}{2}\mu^{i}(t)+\epsilon^{n-1}\int_{\partial \Omega^{o}}\nu_{\omega^{i}}(t)\cdot \nabla S_{n}(\epsilon t-y)\mu^{o}(y)\;d\sigma_{y}+\int_{\partial \omega^{i}}\nu_{\omega^{i}}(t)\cdot \nabla S_{n}(t-s)\mu^{i}(s)\;d\sigma_{s}\\ & \;=\tilde{F}(\epsilon^{n-1}\delta (\epsilon )\int_{\partial \Omega^{o}}S_{n}(\epsilon t-y)\mu^{o}(y)\;d\sigma_{y}+\epsilon \delta (\epsilon )\int_{\partial \omega^{i}}S_{n}(t-s)\mu^{i}(s)\;d\sigma_{s}+\xi ,\eta (\epsilon ))\\ & \;\;+g^{i}(t)\frac{\epsilon^{n-1}}{\rho (\epsilon )}\;\forall \;t\in \partial \omega^{i}, \end{array}$$

for all $\epsilon \in (0,\epsilon_{1})$. We would like to pass to the limit as ϵ→0 in equations (4.1) and (4.2). However, to do so, we need to know the asymptotic behaviour for ϵ close to 0 of the quantities ϵδ(ϵ) and $\epsilon^{n-1}\rho (\epsilon ),$ which appear in (4.2). Accordingly, we now assume that

4.3 $$d_{0}\equiv \lim_{\epsilon \to 0}\epsilon \delta (\epsilon )\in R\;\text{and}\;r_{0}\equiv \lim_{\epsilon \to 0}\frac{\epsilon^{n-1}}{\rho (\epsilon )}\in R.$$

Motivated by (4.1) and (4.2), we replace the quantities ϵδ(ϵ), η(ϵ) and $\epsilon^{n-1}/\rho (\epsilon )$ by the auxiliary variables $\gamma_{1}$, $\gamma_{2}$ and $\gamma_{3}$, respectively, and we now introduce the operator $\Lambda_{n}\equiv (\Lambda_{n}^{o},\Lambda_{n}^{i})$ from $(-\epsilon_{1},\epsilon_{1})\times R^{m+2}\times C^{0,\alpha }{\left(\partial \Omega^{o}\right)}_{0}\times C^{0,\alpha }(\partial \omega^{i})\times R$ to $C^{0,\alpha }(\partial \Omega^{o})\times C^{0,\alpha }(\partial \omega^{i})$ defined by

4.4 $$\begin{array}{rl} & \Lambda_{n}^{o}[\epsilon ,\gamma_{1},\gamma_{2},\gamma_{3},\mu^{o},\mu^{i},\xi ](x)\\ & \;\equiv -\frac{1}{2}\mu^{o}(x)+\int_{\partial \Omega^{o}}\nu_{\Omega^{o}}(x)\cdot \nabla S_{n}(x-y)\mu^{o}(y)\;d\sigma_{y}\\ & \;\;+\int_{\partial \omega^{i}}\nu_{\Omega^{o}}(x)\cdot \nabla S_{n}(x-\epsilon s)\mu^{i}(s)\;d\sigma_{s}-g^{o}(x)\;\forall \;x\in \partial \Omega^{o} \end{array}$$

and

4.5 $$\begin{array}{rl} & \Lambda_{n}^{i}[\epsilon ,\gamma_{1},\gamma_{2},\gamma_{3},\mu^{o},\mu^{i},\xi ](t)\\ & \;\equiv \frac{1}{2}\mu^{i}(t)+\epsilon^{n-1}\int_{\partial \Omega^{o}}\nu_{\omega^{i}}(t)\cdot \nabla S_{n}(\epsilon t-y)\mu^{o}(y)\;d\sigma_{y}+\int_{\partial \omega^{i}}\nu_{\omega^{i}}(t)\cdot \nabla S_{n}(t-s)\mu^{i}(s)\;d\sigma_{s}\\ & \;\;-\tilde{F}(\epsilon^{n-2}\gamma_{1}\int_{\partial \Omega^{o}}S_{n}(\epsilon t-y)\mu^{o}(y)\;d\sigma_{y}+\gamma_{1}\int_{\partial \omega^{i}}S_{n}(t-s)\mu^{i}(s)\;d\sigma_{s}+\xi ,\gamma_{2})\\ & \;\;-g^{i}(t)\gamma_{3}\;\forall \;t\in \partial \omega^{i} \end{array}$$

for all $(\epsilon ,\gamma_{1},\gamma_{2},\gamma_{3},\mu^{o},\mu^{i},\xi )\in (-\epsilon_{1},\epsilon_{1})\times R^{m+2}\times C^{0,\alpha }{\left(\partial \Omega^{o}\right)}_{0}\times C^{0,\alpha }(\partial \omega^{i})\times R$.

Then, if $\epsilon \in (0,\epsilon_{1})$, in view of definitions (4.4) and (4.5), the system of equations

4.6 $$\Lambda_{n}^{o}\left[\epsilon ,\epsilon \delta (\epsilon ),\eta (\epsilon ),\frac{\epsilon^{n-1}}{\rho (\epsilon )},\mu^{o},\mu^{i},\xi \right](x)=0\;\forall \;x\in \partial \Omega^{o}$$

and

4.7 $$\Lambda_{n}^{i}\left[\epsilon ,\epsilon \delta (\epsilon ),\eta (\epsilon ),\frac{\epsilon^{n-1}}{\rho (\epsilon )},\mu^{o},\mu^{i},\xi \right](t)=0\;\forall \;t\in \partial \omega^{i}$$

is equivalent to the system (4.1)–(4.2). Then if we let ϵ→0 in (4.6) and (4.7), we obtain

4.8 $$\begin{array}{rl} & \;-\frac{1}{2}\mu^{o}(x)+\int_{\partial \Omega^{o}}\nu_{\Omega^{o}}(x)\cdot \nabla S_{n}(x-y)\mu^{o}(y)\;d\sigma_{y}\\ & \;\;+\nu_{\Omega^{o}}(x)\cdot \nabla S_{n}(x)\int_{\partial \omega^{i}}\mu^{i}(s)\;d\sigma_{s}=g^{o}(x)\;\forall \;x\in \partial \Omega^{o} \end{array}$$

and

4.9 $$\begin{array}{rl} & \frac{1}{2}\mu^{i}(t)+\int_{\partial \omega^{i}}\nu_{\omega^{i}}(t)\cdot \nabla S_{n}(t-s)\mu^{i}(s)\;d\sigma_{s}\\ & \;=\tilde{F}(d_{0}\int_{\partial \omega^{i}}S_{n}(t-s)\mu^{i}(s)\;d\sigma_{s}+\xi ,\eta_{0})+g^{i}(t)r_{0}\;\forall \;t\in \partial \omega^{i}. \end{array}$$

Now we would like to prove for $\epsilon \in (0,\epsilon_{1})$ the existence of solutions $\left(\mu^{o},\mu^{i},\xi \right)$ to the system (4.6)–(4.7) around a solution of the limiting system (4.8)–(4.9). Therefore, we now further assume that

4.10 $$\begin{array}{rl} & \;\text{the system }(4.8)-(4.9)\text{ in the unknown }(\mu^{o},\mu^{i},\xi )\text{ admits}\\ & \;\text{ a solution }({\tilde{\mu }}^{o},{\tilde{\mu }}^{i},\tilde{\xi })\text{ in }C^{0,\alpha }{\left(\partial \Omega^{o}\right)}_{0}\times C^{0,\alpha }(\partial \omega^{i})\times R. \end{array}$$

We do not discuss here conditions on $\tilde{F}$ ensuring the existence of a solution of system (4.8)–(4.9). However, they can be obtained by arguing as in [32], appendix C, or in [7].

We note that if $\left({\tilde{\mu }}^{o},{\tilde{\mu }}^{i},\tilde{\xi }\right)$ is a solution of the system (4.8)–(4.9), then by integrating (4.8) on $\partial \Omega^{o}$ and using the equalities

$$\int_{\partial \Omega^{o}}\int_{\partial \Omega^{o}}\nu_{\Omega^{o}}(x)\cdot \nabla S_{n}(x-y){\tilde{\mu }}^{o}(y)\;d\sigma_{y}\;d\sigma_{x}=\frac{1}{2}\int_{\partial \Omega^{o}}{\tilde{\mu }}^{o}(y)\;d\sigma_{y}$$

(cf. [31], lemma 6.11) and $\int_{\partial \Omega^{o}}\nu_{\Omega^{o}}(x)\cdot \nabla S_{n}(x)\;d\sigma_{x}=1$ (cf. [31], corollary 4.6), we obtain $\int_{\partial \omega^{i}}{\tilde{\mu }}^{i}(s)\;d\sigma_{s}=\int_{\partial \Omega^{o}}g^{o}(x)\;d\sigma_{x}$. In the following proposition, we investigate the system of integral equations (4.1)–(4.2) by applying the implicit function theorem to $\Lambda_{n}$ under suitable assumptions on $\partial_{\tau }\tilde{F}(d_{0}\int_{\partial \omega^{i}}S_{n}(t-s){\tilde{\mu }}^{i}(s)\;d\sigma_{s}+\tilde{\xi },\eta_{0})$, where $\partial_{\tau }\tilde{F}$ denotes the partial derivative with respect to the variable τ of the function $\left(\tau ,\eta )\mapsto \tilde{F}(\tau ,\eta \right)$.

**Proposition 4.1.**

*Let assumptions* (3.4) *and* (4.3) *hold. Let* $\left({\tilde{\mu }}^{o},{\tilde{\mu }}^{i},\tilde{\xi }\right)$ *be as in* (4.10). *Assume that*

$$\int_{\partial \omega^{i}}\partial_{\tau }\tilde{F}(d_{0}\int_{\partial \omega^{i}}S_{n}(t-s){\tilde{\mu }}^{i}(s)\;d\sigma_{s}+\tilde{\xi },\eta_{0})\;d\sigma_{t}\neq 0$$

*and, if* $d_{0}\neq 0$, *also*

$$\partial_{\tau }\tilde{F}(d_{0}\int_{\partial \omega^{i}}S_{n}(t-s){\tilde{\mu }}^{i}(s)\;d\sigma_{s}+\tilde{\xi },\eta_{0})\geq 0\;\forall \;t\in \partial \omega^{i}.$$

*Then there exist* $\epsilon_{2}\in (0,\epsilon_{1})$, *an open neighbourhood* U of $\left(d_{0},\eta_{0},r_{0}\right)$ *in* $R^{m+2}$, *an open neighbourhood* V *of* $\left({\tilde{\mu }}^{o},{\tilde{\mu }}^{i},\tilde{\xi }\right)$ *in* $C^{0,\alpha }{\left(\partial \Omega^{o}\right)}_{0}\times C^{0,\alpha }(\partial \omega^{i})\times R$ *and a real analytic map* $\left(M^{o},M^{i},\Xi \right)$ *from* $(-\epsilon_{2},\epsilon_{2})\times U$ *to* V *such that*

$$(\epsilon \delta (\epsilon ),\;\eta (\epsilon ),\frac{\epsilon^{n-1}}{\rho (\epsilon )})\in U\;\forall \;\epsilon \in (0,\epsilon_{2})$$

*and such that the set of zeros of* $\Lambda_{n}$ *in* $(-\epsilon_{2},\epsilon_{2})\times U\times V$ *coincides with the graph of* $\left(M^{o},M^{i},\Xi \right)$. *In particular*, $\left(M^{o}[0,d_{0},\eta_{0},r_{0}],M^{i}[0,d_{0},\eta_{0},r_{0}],\Xi [0,d_{0},\eta_{0},r_{0}])=({\tilde{\mu }}^{o},{\tilde{\mu }}^{i},\tilde{\xi }\right)$.

**Proof.**

From standard results of classical potential theory (see e.g. Dalla Riva *et al.* [31], Miranda [49] and Lanza de Cristoforis and Rossi [50]), real analyticity results for integral operators with real analytic kernel (see Lanza de Cristoforis and Musolino [51]), assumption (3.4) and real analyticity results for the composition operator (Böhme and Tomi [52], p. 10, Henry [53] and Valent [54], theorem 5.2), we deduce that $\Lambda_{n}$ is a real analytic operator from $(-\epsilon_{1},\epsilon_{1})\times R^{m+2}\times C^{0,\alpha }{\left(\partial \Omega^{o}\right)}_{0}\times C^{0,\alpha }(\partial \omega^{i})\times R$ to $C^{0,\alpha }(\partial \Omega^{o})\times C^{0,\alpha }(\partial \omega^{i})$. By standard calculus in Banach spaces, we verify that the partial differential $\partial_{\left(\mu^{o},\mu^{i},\xi \right)}\Lambda_{n}[0,d_{0},\eta_{0},r_{0},{\tilde{\mu }}^{o},{\tilde{\mu }}^{i},\tilde{\xi }]$ of $\Lambda_{n}$ at $\left(0,d_{0},\eta_{0},r_{0},{\tilde{\mu }}^{o},{\tilde{\mu }}^{i},\tilde{\xi }\right)$ with respect to the variable $\left(\mu^{o},\mu^{i},\xi \right)$ is given by

$$\begin{array}{rl} & \partial_{\left(\mu^{o},\mu^{i},\xi \right)}\Lambda_{n}^{o}[0,d_{0},\eta_{0},r_{0},{\tilde{\mu }}^{o},{\tilde{\mu }}^{i},\tilde{\xi }]({\bar{\mu }}^{o},{\bar{\mu }}^{i},\bar{\xi })(x)\\ & \;\equiv -\frac{1}{2}{\bar{\mu }}^{o}(x)+\int_{\partial \Omega^{o}}\nu_{\Omega^{o}}(x)\cdot \nabla S_{n}(x-y){\bar{\mu }}^{o}(y)\;d\sigma_{y}+\nu_{\Omega^{o}}(x)\cdot \nabla S_{n}(x)\int_{\partial \omega^{i}}{\bar{\mu }}^{i}(s)\;d\sigma_{s}\;\forall \;x\in \partial \Omega^{o} \end{array}$$

and

$$\begin{array}{rl} & \partial_{\left(\mu^{o},\mu^{i},\xi \right)}\Lambda_{n}^{i}[0,d_{0},\eta_{0},r_{0},{\tilde{\mu }}^{o},{\tilde{\mu }}^{i},\tilde{\xi }]({\bar{\mu }}^{o},{\bar{\mu }}^{i},\bar{\xi })(t)\\ & \;\equiv \frac{1}{2}{\bar{\mu }}^{i}(t)+\int_{\partial \omega^{i}}\nu_{\omega^{i}}(t)\cdot \nabla S_{n}(t-s){\bar{\mu }}^{i}(s)\;d\sigma_{s}\\ & \;\;-\partial_{\tau }\tilde{F}(d_{0}\int_{\partial \omega^{i}}S_{n}(t-s){\tilde{\mu }}^{i}(s)\;d\sigma_{s}+\tilde{\xi },\eta_{0})(d_{0}\int_{\partial \omega^{i}}S_{n}(t-s){\bar{\mu }}^{i}(s)\;d\sigma_{s}+\bar{\xi })\;\forall \;t\in \partial \omega^{i} \end{array}$$

for all $({\bar{\mu }}^{o},{\bar{\mu }}^{i},\bar{\xi })\in C^{0,\alpha }{\left(\partial \Omega^{o}\right)}_{0}\times C^{0,\alpha }(\partial \omega^{i})\times R$. Now we want to show that the partial differential $\partial_{\left(\mu^{o},\mu^{i},\xi \right)}\Lambda_{n}[0,d_{0},\eta_{0},r_{0},{\tilde{\mu }}^{o},{\tilde{\mu }}^{i},\tilde{\xi }]$ is a homeomorphism from $C^{0,\alpha }{\left(\partial \Omega^{o}\right)}_{0}\times C^{0,\alpha }(\partial \omega^{i})\times R$ onto $C^{0,\alpha }(\partial \Omega^{o})\times C^{0,\alpha }(\partial \omega^{i})$. Since $\partial_{\left(\mu^{o},\mu^{i},\xi \right)}\Lambda_{n}[0,d_{0},\eta_{0},r_{0},{\tilde{\mu }}^{o},{\tilde{\mu }}^{i},\tilde{\xi }]$ is the sum of an invertible operator and a compact operator, one immediately verifies that it is a Fredholm operator of index 0. Therefore, to prove that the operator $\partial_{\left(\mu^{o},\mu^{i},\xi \right)}\Lambda_{n}[0,d_{0},\eta_{0},r_{0},{\tilde{\mu }}^{o},{\tilde{\mu }}^{i},\tilde{\xi }]$ is a homeomorphism, it suffices to prove that it is injective. So let us assume that

$$\partial_{\left(\mu^{o},\mu^{i},\xi \right)}\Lambda_{n}[0,d_{0},\eta_{0},r_{0},{\tilde{\mu }}^{o},{\tilde{\mu }}^{i},\tilde{\xi }]({\bar{\mu }}^{o},{\bar{\mu }}^{i},\bar{\xi })=0.$$

By integrating on the $\partial \Omega^{o}$ equality

$$\partial_{\left(\mu^{o},\mu^{i},\xi \right)}\Lambda_{n}^{o}[0,d_{0},\eta_{0},r_{0},{\tilde{\mu }}^{o},{\tilde{\mu }}^{i},\tilde{\xi }]({\bar{\mu }}^{o},{\bar{\mu }}^{i},\bar{\xi })(x)=0\;\forall \;x\in \partial \Omega^{o}$$

and using the equalities

$$\int_{\partial \Omega^{o}}\int_{\partial \Omega^{o}}\nu_{\Omega^{o}}(x)\cdot \nabla S_{n}(x-y){\bar{\mu }}^{o}(y)\;d\sigma_{y}\;d\sigma_{x}=\frac{1}{2}\int_{\partial \Omega^{o}}{\bar{\mu }}^{o}(y)\;d\sigma_{y}$$

(cf. [31], lemma 6.11) and $\int_{\partial \Omega^{o}}\nu_{\Omega^{o}}(x)\cdot \nabla S_{n}(x)\;d\sigma_{x}=1\;$ (cf. [31], corollary 4.6), we obtain

4.11 $$\int_{\partial \omega^{i}}{\bar{\mu }}^{i}(s)\;d\sigma_{s}=0.$$

As a consequence,

$$-\frac{1}{2}{\bar{\mu }}^{o}(x)+\int_{\partial \Omega^{o}}\nu_{\Omega^{o}}(x)\cdot \nabla S_{n}(x-y){\bar{\mu }}^{o}(y)\;d\sigma_{y}=0\;\forall \;x\in \partial \Omega^{o},$$

and thus by [31], theorem 6.25, since $\int_{\partial \Omega^{o}}{\bar{\mu }}^{o}\;d\sigma =0$ we have ${\bar{\mu }}^{o}=0$. By (4.11) and the same argument as in [33], proof of theorem 4.4, the equality

$$\partial_{\left(\mu^{o},\mu^{i},\xi \right)}\Lambda_{n}^{i}[0,d_{0},\eta_{0},r_{0},{\tilde{\mu }}^{o},{\tilde{\mu }}^{i},\tilde{\xi }]({\bar{\mu }}^{o},{\bar{\mu }}^{i},\bar{\xi })(t)=0\;\forall \;t\in \partial \omega^{i}$$

implies that $({\bar{\mu }}^{i},\bar{\xi })=0$. In conclusion, we have shown that $\partial_{\left(\mu^{o},\mu^{i},\xi \right)}\Lambda_{n}[0,d_{0},\eta_{0},r_{0},{\tilde{\mu }}^{o},{\tilde{\mu }}^{i},\tilde{\xi }]$ is injective and thus, being a Fredholm operator of index 0, also a homeomorphism. As a consequence, we can apply the implicit function theorem for real analytic maps in Banach spaces (cf. [55], theorem 15.3) and deduce that there exist $\epsilon_{2}\in (0,\epsilon_{1})$, an open neighbourhood U of $\left(d_{0},\eta_{0},r_{0}\right)$ in $R^{m+2}$, an open neighbourhood V of $\left({\tilde{\mu }}^{o},{\tilde{\mu }}^{i},\tilde{\xi }\right)$ in $C^{0,\alpha }{\left(\partial \Omega^{o}\right)}_{0}\times C^{0,\alpha }(\partial \omega^{i})\times R$ and a real analytic map $\left(M^{o},M^{i},\Xi \right)$ from $(-\epsilon_{2},\epsilon_{2})\times U$ to V with $(\epsilon \delta (\epsilon ),\eta (\epsilon ),(\epsilon^{n-1}/\rho (\epsilon )))\in U$ for all $\epsilon \in (0,\epsilon_{2})$ such that the set of zeros of $\Lambda_{n}$ in $(-\epsilon_{2},\epsilon_{2})\times U\times V$ coincides with the graph of $\left(M^{o},M^{i},\Xi \right)$ and, in particular, $\left(M^{o}[0,d_{0},\eta_{0},r_{0}],M^{i}[0,d_{0},\eta_{0},r_{0}],\Xi [0,d_{0},\eta_{0},r_{0}])=({\tilde{\mu }}^{o},{\tilde{\mu }}^{i},\tilde{\xi }\right)$.

**Remark 4.2.**

If F is linear, system (4.8)–(4.9) simplifies to

4.12 $$\begin{array}{rl} & \;-\frac{1}{2}\mu^{o}(x)+\int_{\partial \Omega^{o}}\nu_{\Omega^{o}}(x)\cdot \nabla S_{n}(x-y)\mu^{o}(y)\;d\sigma_{y}\\ & \;\;+\nu_{\Omega^{o}}(x)\cdot \nabla S_{n}(x)\int_{\partial \omega^{i}}\mu^{i}(s)\;d\sigma_{s}=g^{o}(x)\;\forall \;x\in \partial \Omega^{o} \end{array}$$

and

4.13 $$\begin{array}{rl} & \frac{1}{2}\mu^{i}(t)+\int_{\partial \omega^{i}}\nu_{\omega^{i}}(t)\cdot \nabla S_{n}(t-s)\mu^{i}(s)\;d\sigma_{s}\\ & \;\;=d_{0}\int_{\partial \omega^{i}}S_{n}(t-s)\mu^{i}(s)\;d\sigma_{s}+\xi +g^{i}(t)r_{0}\;\forall \;t\in \partial \omega^{i}. \end{array}$$

Then, by arguing as in the proof of proposition 4.1, one verifies that system (4.12)–(4.13) in the unknown $\left(\mu^{o},\mu^{i},\xi \right)$ admits a unique solution $\left({\tilde{\mu }}^{o},{\tilde{\mu }}^{i},\tilde{\xi }\right)$ in $C^{0,\alpha }{\left(\partial \Omega^{o}\right)}_{0}\times C^{0,\alpha }(\partial \omega^{i})\times R$. By integrating (4.12) and (4.13), we deduce that

$$\frac{1}{\int_{\partial \omega^{i}}\;d\sigma }(\int_{\partial \Omega^{o}}g^{o}\;d\sigma -d_{0}\int_{\partial \omega^{i}}\int_{\partial \omega^{i}}S_{n}(t-s){\tilde{\mu }}^{i}(s)\;d\sigma_{s}\;d\sigma_{t}-r_{0}\int_{\partial \omega^{i}}g^{i}\;d\sigma )=\tilde{\xi }.$$

If we further assume that

$$\Omega^{o}=\omega^{i}=B_{n}(0,1),\;g^{o}(x)=a\;\forall x\in \partial B_{n}(0,1)\;\text{and}\;g^{i}(t)=b\;\forall t\in \partial B_{n}(0,1)$$

for some constants a,b∈R, then by the well-known identity

$$\int_{\partial B_{n}(0,1)}S_{n}(t-s)\;d\sigma_{t}=\frac{1}{2-n}\;\forall s\in \partial B_{n}(0,1),$$

one obtains

$$\frac{1}{s_{n}}(as_{n}-d_{0}\frac{1}{2-n}as_{n}-bs_{n}r_{0})=\tilde{\xi }$$

and thus

$$\tilde{\xi }=a-br_{0}+\frac{a}{n-2}d_{0}.$$

Now that we have converted (1.3) into a system of integral equations for which we have exhibited a real analytic family of solutions, we introduce a family of solutions to (1.3).

**Definition 4.3.**

Let the assumptions of proposition 4.1 hold. Then we set

$$\begin{array}{rl} u(\epsilon ,x) & \;=\int_{\partial \Omega^{o}}S_{n}(x-y)\;M^{o}\left[\epsilon ,\epsilon \delta (\epsilon ),\eta (\epsilon ),\frac{\epsilon^{n-1}}{\rho (\epsilon )}\right](y)\;d\sigma_{y}\\ & \;\;+\int_{\partial \omega^{i}}S_{n}(x-\epsilon s)\;M^{i}\left[\epsilon ,\epsilon \delta (\epsilon ),\eta (\epsilon ),\frac{\epsilon^{n-1}}{\rho (\epsilon )}\right](s)\;d\sigma_{s}\\ & \;\;+\frac{\Xi [\epsilon ,\epsilon \delta (\epsilon ),\eta (\epsilon ),(\epsilon^{n-1}/\rho (\epsilon ))]}{\delta (\epsilon )\epsilon^{n-1}}\;\forall \;x\in \bar{\Omega (\epsilon )},\;\forall \epsilon \in (0,\epsilon_{2}). \end{array}$$

By propositions 3.2 and 4.1 and definition 4.3, we deduce that for each $\epsilon \in (0,\epsilon_{2})$ the function $u(\epsilon ,\cdot )\in C^{1,\alpha }(\bar{\Omega (\epsilon )})$ is a solution to problem (1.3). In the following theorems, we exploit the analyticity result of proposition 4.1 to prove representation formulas for u(ϵ,⋅) and for its energy integral in terms of real analytic maps. We start with the following theorem, which considers the restriction of the solution u(ϵ,⋅) to a set that is ‘far’ from the hole.

**Theorem 4.4.**

*Let the assumptions of proposition 4.1 hold. Let* $\Omega_{M}$ *be a bounded open subset of* $\Omega^{o}$ *such that* $0\notin \bar{\Omega_{M}}$. *Then there exist* $\epsilon_{M}\in (0,\epsilon_{2})$ *and a real analytic map* $U_{M}$ *from* $(-\epsilon_{M},\epsilon_{M})\times U$ *to* $C^{1,\alpha }(\bar{\Omega_{M}})$ *such that* $\bar{\Omega_{M}}\subseteq \bar{\Omega (\epsilon )}$ *for all* $\epsilon \in (0,\epsilon_{M})$ *and*

4.14 $$u(\epsilon ,x)=U_{M}\left[\epsilon ,\epsilon \delta (\epsilon ),\eta (\epsilon ),\frac{\epsilon^{n-1}}{\rho (\epsilon )}\right](x)+\frac{\Xi [\epsilon ,\epsilon \delta (\epsilon ),\eta (\epsilon ),(\epsilon^{n-1}/\rho (\epsilon ))]}{\delta (\epsilon )\epsilon^{n-1}}\;\forall \;x\in \bar{\Omega_{M}}$$

*for all* $\epsilon \in (0,\epsilon_{M})$. *Moreover, if we set*

$${\tilde{u}}_{M}(x)\equiv \int_{\partial \Omega^{o}}S_{n}(x-y){\tilde{\mu }}^{o}(y)\;d\sigma_{y}\;\forall \;x\in \bar{\Omega^{o}},$$

*we have that* $U_{M}[0,d_{0},\eta_{0},r_{0}]={\tilde{u}}_{M|\bar{\Omega_{M}}}+S_{n|\bar{\Omega_{M}}}\int_{\partial \Omega^{o}}g^{o}\;d\sigma$, *and* ${\tilde{u}}_{M}$ *solves the Neumann problem*

4.15 $$\left\{ \begin{array}{ll} \Delta u(x)=0 & \forall \;x\in \Omega^{o},\\ \frac{\partial }{\partial \nu_{\Omega^{o}}}u(x)=g^{o}(x)-\frac{\partial }{\partial \nu_{\Omega^{o}}}S_{n}(x)\underset{\partial \Omega^{o}}{\int }g^{o}\;d\sigma & \forall \;x\in \partial \Omega^{o}. \end{array}\right.$$

**Proof.**

Taking $\epsilon_{M}\in (0,\epsilon_{2})$ small enough, we can assume that $\bar{\Omega_{M}}\cap \epsilon \bar{\omega^{i}}=\emptyset$ for all $\epsilon \in (-\epsilon_{M},\epsilon_{M})$. In view of definition 4.3, it is natural to set

$$\begin{array}{rl} U_{M}[\epsilon ,\gamma_{1},\gamma_{2},\gamma_{3}](x) & \;\equiv \int_{\partial \Omega^{o}}S_{n}(x-y)M^{o}[\epsilon ,\gamma_{1},\gamma_{2},\gamma_{3}](y)\;d\sigma_{y}\\ & \;\;+\int_{\partial \omega^{i}}S_{n}(x-\epsilon s)M^{i}[\epsilon ,\gamma_{1},\gamma_{2},\gamma_{3}](s)\;d\sigma_{s}\;\forall \;x\in \bar{\Omega_{M}}, \end{array}$$

for all $(\epsilon ,\gamma_{1},\gamma_{2},\gamma_{3})\in (-\epsilon_{M},\epsilon_{M})\times U$. By proposition 4.1 and real analyticity results for integral operators with real analytic kernel (cf. [51]), we verify that $U_{M}$ is a real analytic map from $(-\epsilon_{M},\epsilon_{M})\times U$ to $C^{1,\alpha }(\bar{\Omega_{M}})$ and that equality (4.14) holds. By proposition 4.1, we also deduce that $U_{M}[0,d_{0},\eta_{0},r_{0}]={\tilde{u}}_{M|\bar{\Omega_{M}}}+S_{n|\bar{\Omega_{M}}}\int_{\partial \Omega^{o}}g^{o}\;d\sigma$ and, by standard properties of the single-layer potential (cf. [31], §4.4), that ${\tilde{u}}_{M}$ is a solution of problem (4.15). The proof is complete.

**Remark 4.5.**

By proposition 4.1, remark 4.2 and theorem 4.4, if F is linear and

$$\tilde{\xi }=\frac{1}{\int_{\partial \omega^{i}}\;d\sigma }(\int_{\partial \Omega^{o}}g^{o}\;d\sigma -d_{0}\int_{\partial \omega^{i}}\int_{\partial \omega^{i}}S_{n}(t-s){\tilde{\mu }}^{i}(s)\;d\sigma_{s}\;d\sigma_{t}-r_{0}\int_{\partial \omega^{i}}g^{i}\;d\sigma )\neq 0,$$

we deduce that the value of the solution at a fixed point $\bar{x}\in \bar{\Omega^{o}}\setminus \{0\}$ is asymptotic to

$$\frac{1/(\int_{\partial \omega^{i}}d\sigma )\left(\int_{\partial \Omega^{o}}g^{o}\;d\sigma -d_{0}\int_{\partial \omega^{i}}\int_{\partial \omega^{i}}S_{n}(t-s){\tilde{\mu }}^{i}(s)\;d\sigma_{s}\;d\sigma_{t}-r_{0}\int_{\partial \omega^{i}}g^{i}\;d\sigma \right)}{\delta (\epsilon )\epsilon^{n-1}}\;\;\text{as}\;\epsilon \to 0.$$

If we further assume that

$$\Omega^{o}=\omega^{i}=B_{n}(0,1),\;g^{o}(x)=a\;\forall x\in \partial B_{n}(0,1)\;\text{and}\;g^{i}(t)=b\;\forall t\in \partial B_{n}(0,1)$$

for some constants a,b∈R, then if

$$a-br_{0}+\frac{a}{n-2}d_{0}\neq 0,$$

we deduce that the value of the solution at a fixed point $\bar{x}\in \bar{B_{n}(0,1)}\setminus \{0\}$ is asymptotic to

$$\frac{a-br_{0}+a\;d_{0}/(n-2)}{\delta (\epsilon )\epsilon^{n-1}}\;\;\text{as}\;\epsilon \to 0.$$

Thus, we recover the result of §2 on the toy problem.

We now consider in theorem 4.6 the behaviour of the rescaled solution u(ϵ,ϵt).

**Theorem 4.6.**

*Let the assumptions of proposition 4.1 hold. Let* $\Omega_{m}$ *be a bounded open subset of* $R^{n}\setminus \bar{\omega^{i}}$. *Then there exist* $\epsilon_{m}\in (0,\epsilon_{2})$ *and a real analytic map* $U_{m}$ *from* $(-\epsilon_{m},\epsilon_{m})\times U$ *to* $C^{1,\alpha }(\bar{\Omega_{m}})$ *such that* $\epsilon \bar{\Omega_{m}}\subseteq \bar{\Omega (\epsilon )}$ *for all* $\epsilon \in (0,\epsilon_{m})$ *and*

$$u(\epsilon ,\epsilon t)=\frac{1}{\epsilon^{n-2}}U_{m}\left[\epsilon ,\epsilon \delta (\epsilon ),\eta (\epsilon ),\frac{\epsilon^{n-1}}{\rho (\epsilon )}\right](t)+\frac{\Xi [\epsilon ,\epsilon \delta (\epsilon ),\eta (\epsilon ),(\epsilon^{n-1}/\rho (\epsilon ))]}{\delta (\epsilon )\epsilon^{n-1}}\;\forall \;x\in \bar{\Omega_{m}}$$

*for all* $\epsilon \in (0,\epsilon_{m})$. *Moreover, if we set*

$${\tilde{u}}_{m}(t)\equiv \int_{\partial \omega^{i}}S_{n}(t-s){\tilde{\mu }}^{i}(s)\;d\sigma_{s}\;\forall \;t\in R^{n}\setminus \omega^{i},$$

*we have that* $U_{m}[0,d_{0},\eta_{0},r_{0}]={\tilde{u}}_{m|\bar{\Omega_{m}}}$ *and* ${\tilde{u}}_{m}$ *solves the (nonlinear) Robin problem*

4.16 $$\left\{ \begin{array}{ll} \Delta u(t)=0 & \forall \;t\in R^{n}\setminus \bar{\omega^{i}},\\ \frac{\partial }{\partial \nu_{\omega^{i}}}u(t)=\tilde{F}(d_{0}u(t)+\tilde{\xi },\eta_{0})+g^{i}(t)r_{0} & \forall \;t\in \partial \omega^{i},\\ \lim_{t\to \infty }u(t)=0. & \end{array}\right.$$

**Proof.**

Taking $\epsilon_{m}\in (0,\epsilon_{2})$ small enough, we can assume that $\epsilon \bar{\Omega_{m}}\subseteq \bar{\Omega^{o}}$ for all $\epsilon \in (-\epsilon_{m},\epsilon_{m})$. By definition 4.3, we note that if $\epsilon \in (0,\epsilon_{m})$, then

$$\begin{array}{rl} u(\epsilon ,\epsilon t) & \;=\int_{\partial \Omega^{o}}S_{n}(\epsilon t-y)M^{o}\left[\epsilon ,\epsilon \delta (\epsilon ),\eta (\epsilon ),\frac{\epsilon^{n-1}}{\rho (\epsilon )}\right](y)\;d\sigma_{y}\\ & \;\;+\int_{\partial \omega^{i}}S_{n}(\epsilon t-\epsilon s)M^{i}\left[\epsilon ,\epsilon \delta (\epsilon ),\eta (\epsilon ),\frac{\epsilon^{n-1}}{\rho (\epsilon )}\right](s)\;d\sigma_{s}+\frac{\Xi [\epsilon ,\epsilon \delta (\epsilon ),\eta (\epsilon ),(\epsilon^{n-1}/\rho (\epsilon ))]}{\delta (\epsilon )\epsilon^{n-1}}\\ & \;\;=\frac{1}{\epsilon^{n-2}}(\epsilon^{n-2}\int_{\partial \Omega^{o}}S_{n}(\epsilon t-y)M^{o}\left[\epsilon ,\epsilon \delta (\epsilon ),\eta (\epsilon ),\frac{\epsilon^{n-1}}{\rho (\epsilon )}\right](y)\;d\sigma_{y}\\ & \;\;+\int_{\partial \omega^{i}}S_{n}(t-s)M^{i}\left[\epsilon ,\epsilon \delta (\epsilon ),\eta (\epsilon ),\frac{\epsilon^{n-1}}{\rho (\epsilon )}\right](s)\;d\sigma_{s})+\frac{\Xi [\epsilon ,\epsilon \delta (\epsilon ),\eta (\epsilon ),(\epsilon^{n-1}/\rho (\epsilon ))]}{\delta (\epsilon )\epsilon^{n-1}}\;\forall \;t\in \bar{\Omega_{m}}. \end{array}$$

Accordingly, we set

$$\begin{array}{rl} U_{m}[\epsilon ,\gamma_{1},\gamma_{2},\gamma_{3}](t) & \;\equiv \epsilon^{n-2}\int_{\partial \Omega^{o}}S_{n}(\epsilon t-y)M^{o}[\epsilon ,\gamma_{1},\gamma_{2},\gamma_{3}](y)\;d\sigma_{y}\\ & \;\;+\int_{\partial \omega^{i}}S_{n}(t-s)M^{i}[\epsilon ,\gamma_{1},\gamma_{2},\gamma_{3}](s)\;d\sigma_{s}\;\forall \;t\in \bar{\Omega_{m}}, \end{array}$$

for all $(\epsilon ,\gamma_{1},\gamma_{2},\gamma_{3})\in (-\epsilon_{m},\epsilon_{m})\times U$. By proposition 4.1 and real analyticity results for integral operators with real analytic kernel (cf. [51]), we verify that $U_{m}$ is a real analytic map from $(-\epsilon_{m},\epsilon_{m})\times U$ to $C^{1,\alpha }(\bar{\Omega_{m}})$ and that equality (4.14) holds. By proposition 4.1, we also deduce that $U_{m}[0,d_{0},\eta_{0},r_{0}]={\tilde{u}}_{m|\bar{\Omega_{m}}}$ and, by standard properties of the single-layer potential (cf. [31], §4.4), that ${\tilde{u}}_{m}$ is a solution of the (nonlinear) Robin problem (4.16). The proof is complete.

Finally, we consider the energy integral $\int_{\Omega (\epsilon )}|\nabla u(\epsilon ,x){|}^{2}\;dx$ when ϵ is close to 0.

**Theorem 4.7.**

*Let the assumptions of proposition 4.1 hold. Let* ${\tilde{u}}_{m}$ *be as in theorem 4.6. Then there exist* $\epsilon_{e}\in (0,\epsilon_{2})$ *and a real analytic map* E *from* $(-\epsilon_{e},\epsilon_{e})\times U$ *to* R *such that*

4.17 $$\int_{\Omega (\epsilon )}|\nabla u(\epsilon ,x){|}^{2}\;dx=\frac{1}{\epsilon^{n-2}}\;E\left[\epsilon ,\epsilon \delta (\epsilon ),\eta (\epsilon ),\frac{\epsilon^{n-1}}{\rho (\epsilon )}\right]$$

*for all* $\epsilon \in (0,\epsilon_{e})$. *Moreover*,

4.18 $$E[0,d_{0},\eta_{0},r_{0}]=\int_{R^{n}\setminus \omega^{i}}|\nabla {\tilde{u}}_{m}(t){|}^{2}\;dt.$$

**Proof.**

Let $\epsilon \in (0,\epsilon_{1})$. By the divergence theorem, we have that

$$\begin{array}{rl} \int_{\Omega (\epsilon )}|\nabla u(\epsilon ,x){|}^{2}\;dx & \;=\int_{\partial \Omega^{o}}u(\epsilon ,x)\frac{\partial }{\partial \nu_{\Omega^{o}}}u(\epsilon ,x)\;d\sigma_{x}-\int_{\partial \epsilon \omega^{i}}u(\epsilon ,x)\frac{\partial }{\partial \nu_{\epsilon \omega^{i}}}u(\epsilon ,x)\;d\sigma_{x}\\ & \;=\int_{\partial \Omega^{o}}u(\epsilon ,x)\frac{\partial }{\partial \nu_{\Omega^{o}}}u(\epsilon ,x)\;d\sigma_{x}-\epsilon^{n-1}\int_{\partial \omega^{i}}u(\epsilon ,\epsilon t)\nu_{\omega^{i}}(t)\cdot \nabla u(\epsilon ,\epsilon t)\;d\sigma_{t}. \end{array}$$

Then, let $U_{M}$ and $\epsilon_{M}$ be as in theorem 4.4, with $\Omega_{M}\equiv \Omega^{o}\setminus \bar{B_{n}(0,r_{M})}$ for some $r_{M}>0$ such that $\bar{B_{n}(0,r_{M})}\subseteq \Omega^{o}$. Then one verifies that if $\epsilon \in (0,\epsilon_{M}),$

$$\begin{array}{rl} & \;\int_{\partial \Omega^{o}}u(\epsilon ,x)\frac{\partial }{\partial \nu_{\Omega^{o}}}u(\epsilon ,x)\;d\sigma_{x}\\ & \;\;=\int_{\partial \Omega^{o}}U_{M}\left[\epsilon ,\epsilon \delta (\epsilon ),\eta (\epsilon ),\frac{\epsilon^{n-1}}{\rho (\epsilon )}\right](x)\nu_{\Omega^{o}}(x)\cdot \nabla U_{M}\left[\epsilon ,\epsilon \delta (\epsilon ),\eta (\epsilon ),\frac{\epsilon^{n-1}}{\rho (\epsilon )}\right](x)\;d\sigma_{x}. \end{array}$$

Then, let $U_{m}$ and $\epsilon_{m}$ be as in theorem 4.6, with $\Omega_{m}\equiv B_{n}(0,r_{m})\setminus \bar{\omega^{i}}$ for some $r_{m}>0$ such that $B_{n}(0,r_{m})⊇\bar{\omega^{i}}$. Then one verifies that if $\epsilon \in (0,\epsilon_{m}),$

$$\begin{array}{rl} & \epsilon^{n-1}\int_{\partial \omega^{i}}u(\epsilon ,\epsilon t)\nu_{\omega^{i}}(t)\cdot \nabla u(\epsilon ,\epsilon t)\;d\sigma_{t}\\ & \;\;=\frac{1}{\epsilon^{n-2}}\int_{\partial \omega^{i}}U_{m}\left[\epsilon ,\epsilon \delta (\epsilon ),\eta (\epsilon ),\frac{\epsilon^{n-1}}{\rho (\epsilon )}\right](t)\nu_{\omega^{i}}(t)\cdot \nabla U_{m}\left[\epsilon ,\epsilon \delta (\epsilon ),\eta (\epsilon ),\frac{\epsilon^{n-1}}{\rho (\epsilon )}\right](t)\;d\sigma_{t}. \end{array}$$

As a consequence, we set $\epsilon_{e}\equiv \min\{\epsilon_{m},\epsilon_{M}\}$ and

$$\begin{array}{rl} E[\epsilon ,\gamma_{1},\gamma_{2},\gamma_{3}] & \;\equiv \epsilon^{n-2}\int_{\partial \Omega^{o}}U_{M}[\epsilon ,\gamma_{1},\gamma_{2},\gamma_{3}](x)\nu_{\Omega^{o}}(x)\cdot \nabla U_{M}[\epsilon ,\gamma_{1},\gamma_{2},\gamma_{3}](x)\;d\sigma_{x}\\ & \;\;-\int_{\partial \omega^{i}}U_{m}[\epsilon ,\gamma_{1},\gamma_{2},\gamma_{3}](t)\nu_{\omega^{i}}(t)\cdot \nabla U_{m}[\epsilon ,\gamma_{1},\gamma_{2},\gamma_{3}](t)\;d\sigma_{t} \end{array}$$

for all $(\epsilon ,\gamma_{1},\gamma_{2},\gamma_{3})\in (-\epsilon_{e},\epsilon_{e})\times U$. We verify that E is a real analytic map from $(-\epsilon_{e},\epsilon_{e})\times U$ to R and that equality (4.17) holds. Moreover, by the behaviour at infinity of ${\tilde{u}}_{m}$ and the divergence theorem on exterior domains (cf. [31], §§3.4 and 4.2), we verify that

$$E[0,d_{0},\eta_{0},r_{0}]=-\int_{\partial \omega^{i}}{\tilde{u}}_{m}(t)\nu_{\omega^{i}}(t)\cdot \nabla {\tilde{u}}_{m}(t)\;d\sigma_{t}=\int_{R^{n}\setminus \omega^{i}}|\nabla {\tilde{u}}_{m}(t){|}^{2}\;dt,$$

and accordingly equality (4.18) holds.

## 5. Conclusion

We have studied the asymptotic behaviour of solutions of a boundary value problem for the Laplace equation in a perforated domain of $R^{n}$, n≥3, with a (nonlinear) Robin boundary condition which may degenerate into a Neumann condition on the boundary of a small hole of size ϵ. Under suitable assumptions, for ϵ close to 0, the value of the solution at a fixed point far from the origin behaves as $1/(\delta (\epsilon )\epsilon^{n-1})$, where δ(ϵ) is a coefficient of a nonlinear function of the trace of the solution in the Robin boundary condition. We have also investigated the behaviour of the energy integral of the solutions as ϵ tends to 0: the energy integral behaves as $1/\epsilon^{n-2}$ multiplied by the energy integral of a solution of an exterior nonlinear Robin problem. In particular, if $\delta (\epsilon )=\epsilon^{r}$, then to satisfy assumption (4.3) we need to have r≥−1, and we have that the value of the solution at a fixed point behaves as $1/\epsilon^{n-1+r}$, whereas the energy integral behaves as $1/\epsilon^{n-2}$ (and such behaviour is not affected by the specific power $\delta (\epsilon )=\epsilon^{r}$). As we have seen, our study is confined to the case of dimension n≥3. We plan to investigate the two-dimensional case (which requires a different analysis due to the logarithmic behaviour of the fundamental solution) in a forthcoming paper. Moreover, together with the study of the planar case, we wish to include numerical examples.

## Data Availability

This article has no additional data.
